# Phenolic Acid Content and Antioxidant Properties of Extruded Corn Snacks Enriched with Kale

**DOI:** 10.1155/2018/7830546

**Published:** 2018-02-04

**Authors:** Kamila Kasprzak, Tomasz Oniszczuk, Agnieszka Wójtowicz, Monika Waksmundzka-Hajnos, Marta Olech, Renata Nowak, Renata Polak, Anna Oniszczuk

**Affiliations:** ^1^Department of Inorganic Chemistry, Medical University of Lublin, Chodźki 4a, 20-093 Lublin, Poland; ^2^Department of Food Process Engineering, University of Life Sciences in Lublin, Doświadczalna 44, 20-280 Lublin, Poland; ^3^Department of Pharmaceutical Botany, Medical University of Lublin, Chodźki 1, 20-093 Lublin, Poland; ^4^Department of Thermal Technology, University of Life Sciences in Lublin, Doświadczalna 44, 20-280 Lublin, Poland

## Abstract

Prohealth food contains specific components which have positive influence on the health and well-being of the consumer. An important position among bioactive compounds occurs for polyphenols. Many results have indicated that an increased intake of phenolic compounds may reduce the risk of cardiovascular diseases and type 2 diabetes. The objective of the study was production of extruded corn snacks with addition (0, 2, 4, 6, and 8%) of kale (*Brassica oleracea* L. var. *sabellica*)—a polyphenol-rich plant. Afterwards, high-performance liquid chromatography-mass spectrometry (LC-ESI-MS/MS) and antioxidant activity analyses of snack extracts were performed. In the corn snacks enriched with kale, fifteen phenolic acids were indicated. These were protocatechuic, 4-OH-benzoic, vanillic, *trans*-caffeic, *cis*-caffeic, *trans*-p-coumaric, *cis*-p-coumaric, *trans*-ferulic, *cis*-ferulic, salicylic, gentisic, syringic, 3-OH-cinnamic, *trans*-sinapic, and *cis*-sinapic acids. Both the qualitative and quantitative content of polyphenols increased with the addition of *B. oleracea*. Data from spectrophotometric analyses of the samples showed high DPPH radical scavenging potential of snacks enriched with 4, 6, and 8% of kale. Snacks enriched with kale contain high level of phenolic acids and, therefore, have great potential to make a valuable source of natural antioxidants. High-temperature short-time extrusion-cooking process had no negative impact on polyphenol's activity.

## 1. Introduction

Prohealth food (functional food and medical food) contains specific components which have positive influence on the health and well-being of the consumer. The concept of “functional food” originated in the early 1980s in Japan [1]. Food could be considered as functional if it provides disease prevention and health promotion against one or more illnesses apart from its nutritional function [2, 3]. This kind of victuals may contain a wide range of functional ingredients, such as vitamins, mineral supplement, herbs, phytochemicals, and probiotics, that possess positive physiological effects [4, 5]. These components can be added to, naturally enhanced, or modified in a food, because of their diverse health benefits, such as antioxidant, anti-inflammatory, cardioprotective, antidiabetic, and anticancer activities [3].

Secondary metabolites from plants as bioactive compounds impact on health. Among them, polyphenols have been extensively investigated during the last years [6–8]. Many results have indicated that an increased intake of polyphenols may reduce the risk of cardiovascular diseases and type 2 diabetes [9]. These results were obtained from experiments conducted in animal models with physiologically realistic levels of isolated phenolic compounds [10, 11] and in humans consuming polyphenol-rich foods [12]. The consumption of products abundant in polyphenols has been shown as positively influencing on many risk factors for cardiovascular diseases (CVDs), such as low-density lipoprotein (LDL) cholesterol, blood pressure (BP), and endothelial function [13–15].

It is well accepted that fruits and vegetables are important components of a healthy diet and that their consumption helps to prevent a wide range of diseases [16–18]. These beneficial properties have been associated with the presence of bioactive compounds [19, 20]. Curly kale (*Brassica oleracea* L. var. *sabellica*) is a traditional crop which has regained attention due to the increased focus on Nordic food, as well as its health-related potential on account of high concentrations of phytochemicals, such as polyphenols and vitamins [21–23], and to have high antioxidant capacity [24, 25].

The growing burden of CVDs, especially in third world countries, has raised interest for new food sources rich in phenolic compounds. In order to introduce new processes that would contribute to the development of functional foods for improving the health of the general population, numerous investigations were performed over the last decade. Nowadays, researchers develop “new” or “emerging technologies” which modify functional properties of food as well as help intensify existing processes. One of the most interesting techniques of functional food production is HTST (high-temperature short-time) extrusion-cooking [26, 27].

Therefore, the objective of the study reported in presented paper was production of extruded corn snacks supplemented with addition of 2, 4, 6, and 8% of kale (*Brassica oleracea* L. var. *sabellica*)—a polyphenol-rich plant. Afterwards, high-performance liquid chromatography-mass spectrometry (LC-ESI-MS/MS) and antioxidant activity analyses of snack extracts were performed.

## 2. Materials and Methods

### 2.1. Chemicals and Plant Material

Standards of phenolic acids were purchased from Sigma-Aldrich Fine Chemicals (St. Louis, MO, USA). All the chemicals were of analytical grade. LC grade methanol (MeOH) was purchased from J.T. Baker (Phillipsburg, USA). LC grade water was prepared using a Millipore Direct-Q3 purification system (Bedford, MA, USA). *Brassica oleracea* L. var. *sabellica* (kale) was purchased from “Klasa” company (Kurów, Poland). The plant was dried in the convection dryer at an average temperature of 39.0°C. The dry plant material was milled to particle size lower than 500 *μ*m and sieved.

### 2.2. Extrusion-Cooking Procedure

Snacks enriched with kale were produced first time in the Department of Food Process Engineering, University of Life Sciences in Lublin. Corn was used as a control. Blends of corn grits and ground kale were prepared by mixing dry components by replacing corn with 2, 4, 6, and 8% of kale. The samples were conditioned to 15% moisture by spraying with water and mixing continuously for 10 minutes. Blends were processed in a TS-45 single screw extrusion-cooker with *L*/*D* = 12 (ZMCh Metalchem, Gliwice, Poland), according to the previously described method [28]. Snacks were ground (bellow 500 *μ*m) and stored in a dark place in closed plastic bags before tests.

### 2.3. Extraction Procedures

Ultrasound-assisted extraction (UAE) was carried out in an ultrasonic bath (J.P. Selecta, Barcelona, Spain; frequency 20 kHz, power 100 W) with a thermostat. Extraction was performed with 2 g of sample at a temperature of 60°C with 40 mL of solvent in each cycle, by three cycles for 10 min (30 min, previously optimized). Four different solvent systems (ethanol, methanol, 80% aqueous ethanol, and 80% aqueous methanol) were evaluated for the extraction of phenolic acids. The highest yields of all analyzed compounds gave ethanol. After evaporation of the extracts, the residues were dissolved in methanol (10 mL). The whole procedure was repeated three times for each sample [29]. Before chromatographic analysis, the extracts were filtered through a 0.45 *μ*m nylon syringe filter.

### 2.4. LC-ESI-MS/MS Analysis of Phenolic Compounds

According to the method described previously [29], the samples were analyzed by high-performance liquid chromatography and electrospray ionization mass spectrometry (HPLC-ESI-MS/MS). Analysis was performed using Agilent 1200 Series HPLC (Agilent Technologies, USA) equipped with a binary gradient solvent pump, a degasser, an autosampler, and a column oven. Phenolic acids were separated at 25°C, on Zorbax SB-C18 column (2.1 × 50 mm, 1.8 *µ*m particle size; Agilent Technologies, USA), using 3 *µ*L injections. MS detection was performed in a 3200 QTRAP mass spectrometer (AB Sciex, USA) equipped with an electrospray ionization source (ESI) and a triple quadrupole-ion trap mass analyzer that was controlled by the Analyst 1.5 software. The QTRAP-MS system was equipped with an electrospray ionization source (ESI) operated in the negative ion mode. The correlation coefficient of all calibration curves was *R*^2^ > 0.9988. Analytical results and parameters of LC-MS/MS quantitative method—data for calibration curves, limit of detection (LOD), and the limit of quantification (LOQ) values for each analyzed phenolic acids—are described in the paper presented previously [29].

### 2.5. Radical-Scavenging Activity of the Analyzed Extracts

The radical-scavenging activity of the extracts was determined spectrophotometrically against the DPPH radical [30]. The concentration of DPPH used for the experiment was 0.1 mM (4 mg of the free radical in 100 mL of methanol). Measurement of reference sample of DPPH solution was performed by mixing 2.0 mL of the solution and 1.0 mL of methanol. Measurement of snack extracts was done after mixing 2.0 mL of DPPH solution and 1.0 mL of the extracts. Each measurement was repeated three times at the wave length of 517 nm at room temperature. The final result was the average of three replicates. The antioxidant activity was calculated with the following formula [31]:

(1) $$\begin{array}{c} \%\text{ DPPH radical scavenging ability}=\frac{A_{0}-A_{1}}{A_{0}}\times 100\;\left[\%\right], \end{array}$$

where *A*_0_ is the absorbance of the reference sample and *A*_1_ is the absorbance of the sample with tested extracts.

## 3. Results and Discussion

The well-known correlation between nutrition and fine fettle demonstrates the great possibilities of food to maintain or improve people's health. Extremely important point of the research is the extraction and characterization of natural components (such as polyphenols), with biological activity that can be incorporated into a functional food, contributing to consumer's well-being. Previous studies of the authors have found high content of the phenolic compounds in *Brassica oleracea* L. var. *sabellica* (kale) [29]. Taking this fact into account, the authors produced corn snacks enriched with kale using extrusion-cooking process and investigated the phenolic acid content and antioxidant properties of these samples. Extrusion-cooking, as HTST method, seems to be one of the best methods for obtaining the maximum nutritive value of several plant products [32].

The first step of the experiment was extraction of polyphenols from snack samples. The most effective conditions for the isolation of analyzed phenolic acids using UAE were as follows: extraction time 30 min, ultrasound frequency 20 kHz, ultrasound power 100, and ethanol as extractant.

**Figure 1:**
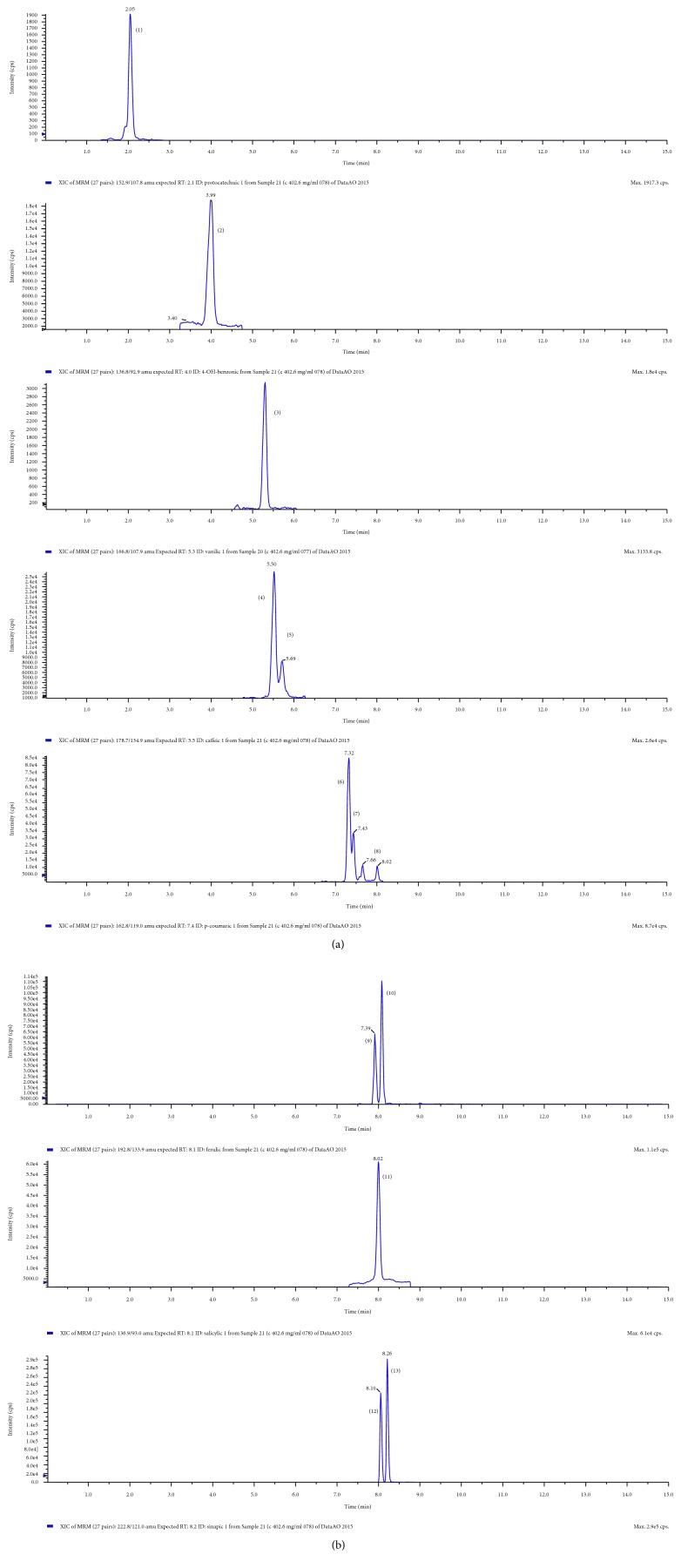
Exemplary LC-ESI-MS/MS chromatogram of analyzed phenolic acids. (1) Protocatechuic acid; (2) 4-OH-benzoic acid; (3) vanilic acid; (4) *trans*-caffeic acid; (5) *cis*-caffeic acid; (6) *trans*-p-coumaric acid; (7) *cis*-p-coumaric acid; (8) 3-OH-cinnamic acid; (9) *trans*-ferulic acid; (10) *cis*-ferulic acid; (11) salicylic acid; (12) *trans*-sinapic acid; (13) *cis*-sinapic acid.

The next point of the study was high-performance liquid chromatography-mass spectrometry analysis of the polyphenolic extracts. In the corn snacks enriched with kale, fifteen phenolic acids were indicated (Table 1). These were protocatechuic, 4-OH-benzoic, vanillic, *trans*-caffeic, *cis*-caffeic, *trans*-p-coumaric, *cis*-p-coumaric, *trans*-ferulic, *cis*-ferulic, salicylic, gentisic, syringic, 3-OH-cinnamic, *trans*-sinapic, and *cis*-sinapic acids. However, concentrations of gentisic and syringic acids were lower than the limit of quantification (LOQ) but higher than the limit of detection (LOD). Snacks without additives contained only eight phenolic acids: 4-OH-benzoic, *trans*-p-coumaric, *cis*-p-coumaric, *trans*-ferulic, *cis*-ferulic, salicylic, *trans*-sinapic and *cis*-sinapic acids. Yields of the following acids were lower than the limit of quantification (LOQ) but higher than the limit of detection (LOD): 4-OH-benzoic, *trans*-sinapic, and *cis*-sinapic. Both the qualitative and quantitative contents of polyphenols increased with the addition of kale; for example, 3-OH-cinnamic acid occurs only in snacks enriched with 6% of *B. oleracea*. Exemplary chromatogram of analyzed phenolic acids is presented in Figure 1.

In order to assess the accuracy of the methods, recovery studies were performed. The samples moistened with the solvent were spiked with known amounts of each standard solution (three concentration levels). Afterwards, ultrasound-assisted extraction was carried out using the same ways employed in the quantitative determination of phenolic compounds in the samples. The recoveries were in the range of 89.2% (for vanillic acid) to 97.3% (for salicylic acid).

**Figure 2:**
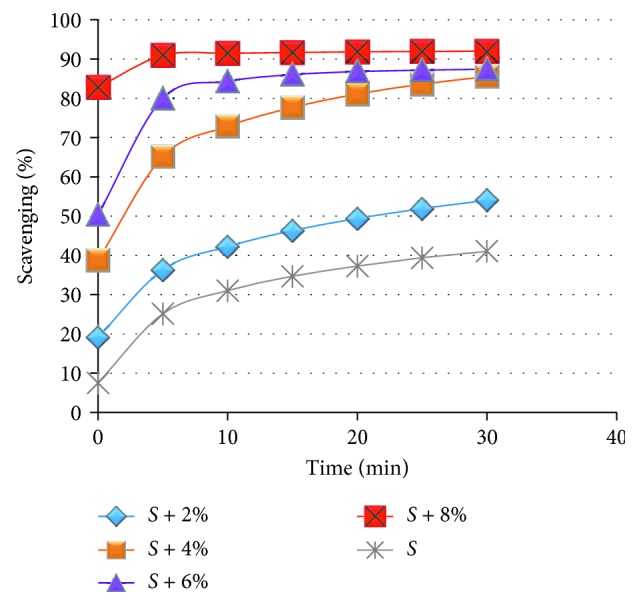
Free radical scavenging activity of extracts of corn snacks (S). Snacks enriched with 2, 4, 6, and 8% kale (*S* + 2%, *S* + 4%, *S* + 6%, and *S* + 8%, resp.) towards DPPH in methanol.

Data from spectrophotometric analyses of the samples showed high DPPH radical- scavenging potential of snacks enriched with 8, 6, and 4% of kale (Figure 2). Antioxidant properties of analyzed extracts were positively correlated with the level of *B. oleracea* in corn snacks. Free radical scavenging ability increased with the addition of kale. The snacks without additives and containing 2% of kale did not scavenge free radical. High antioxidant properties of active samples were observed right now after the first five minutes of the experiment, while maximum radical-scavenging activity was observed after 30 min.

Kale contains a complex mixture of health-related phytochemicals including phenolic compounds [33]. The findings of the research have demonstrated that snacks enriched with kale contain high level of phenolic acids and, therefore, have great potential to be a good source of natural antioxidants. These products have the potential to reduce the risks of lifestyle diseases, for example, chronic inflammation, cardiovascular diseases, and type 2 diabetes, which have become an epidemic and require concerted effort in their treatment. The application of dietary therapy in these disorders is one of the options to fight them. The aim of the experiment conducted by Grace et al. was to test the kale phytoactive compounds (e.g., polyphenols) complexed with proteins as bioactive food ingredients [34]. Biofortified matrices, created with kale, bound to edible proteins were tested for their ability to inhibit biomarkers of acute and chronic inflammation [35]. The matrices demonstrated nutritional and sensory attributes amenable to the use as food component, while delivering anti-inflammatory benefits expected from kale consumption, while delivering anti-inflammatory benefits expected from kale consumption. Results of anti-inflammatory bioassays demonstrated that kale-fortified matrices, at reasonable dietary levels, suppressed the activation of key proinflammatory genes triggered by an inflammatory stimulus [34].

Our results show that starch-based products with high quantity of polyphenols may be produced by extrusion-cooking, because this high-temperature short-time process had no negative impact on polyphenol's activity. These findings are in accordance with the work of Anton et al. [36], who observed a significant increase in total polyphenol content and antioxidant activity of extruded snacks obtained from blends of corn starch and navy/small red beans. Korus et al. [37] studied the effect of extrusion on several polyphenols in raw and extruded beans. Interestingly, they observed an increase of 14% in the amount of phenolics in extrudates compared to raw dark-red beans. The overall increase of 14% observed in dark-red beans during extrusion was mainly due to an increase in quercetin (by 84%) and ferulic acid (by 40%) along with a decrease in chlorogenic and caffeic acids by 33 and 9%, respectively. Thus, extrusion cooking of whole bean flour is advantageous to obtain products with higher levels of polyphenols, provided products poses sensorial acceptance [38]. Other authors [39] reported a significant increase in free/bound phenolic acids such as syringic, ferulic, and coumaric, except vanillic acid during extrusion of buckwheat (*Fagopyrum esculentum* Moench L.). The observed increase in these phenolic compounds could be due to the increased release of these bioactive compounds from the matrix due to extrusion, thus accessible in the extraction.

In conclusion, the benefits associated with regular dietary consumption of phytochemically rich food, for example, corn snacks enriched with kale, contribute to reduced chronic inflammation and can provide anti-infective and other health benefits.

## 4. Conclusions

In the corn snacks enriched with kale, fifteen phenolic acids were indicated. The results of the above study indicate that snacks enriched with kale prepared by the extrusion-cooking process have great potential to be a good source of natural antioxidants with substantiated health benefits, especially when 4% or more was applied. These products have the potential to reduce the risks of lifestyle diseases, for example, cardiovascular diseases and type 2 diabetes. HTST extrusion-cooking processing of snacks had no negative impact on the antioxidant activity of phenolic acids.

## Figures and Tables

**Table 1:** Content of phenolic acids in corn snacks with addition of kale (2, 4, 6, and 8%) (*n* = 3).

Phenolic acid	Yield of phenolic acids from snack samples (*µ*g·g^−1^ of dry weight)				
	Corn snacks	Snacks + 2% kale	Snacks + 4% kale	Snacks + 6% kale	Snacks + 8% kale
Protocatechuic	—	0.054	0.061	0.135	0.193
RSD%	—	2.34	3.66	1.21	0.33
4-OH-Benzoic	BQL	0.114	0.120	0.213	0.248
RSD%	—	0.24	1.78	2.55	3.21
Vanillic	—	0.757	0.810	1.028	1.173
RSD%	—	0.41	1.67	3.46	4.22
*trans*-Caffeic	—	0.218	0.223	0.293	0.374
RSD%	—	4.12	3.45	0.06	0.32
*cis*-Caffeic	—	0.075	0.068	0.103	0.105
RSD%	—	3.67	2.11	0.98	1.34
*trans*-p-Coumaric	0.636	1.062	1.004	1.255	1.246
RSD%	3.34	2.11	2.78	1.82	1.45
*cis*-p-Coumaric	0.013	0.210	0.351	0.439	0.448
RSD%	3.21	4.15	4.21	0.43	2.54
*trans*-Ferulic	0.126	0.599	0.617	1.309	1.690
RSD%	0.12	1.34	1.87	3.24	1.76
*cis*-Ferulic	0.421	1.017	1.928	2.533	2.920
RSD%	3.56	2.24	0.34	0.76	2.45
Salicylic	0.197	0.221	0.267	0.315	0.370
RSD%	4.87	3.25	1.67	4.79	0.34
3-OH-Cinnamic	—	—	—	BQL	0.155
RSD%	—	—	—	—	2.98
*trans*-Sinapic	BQL	2.944	3.335	4.177	5.787
RSD%	—	4.21	3.45	1.31	2.15
*cis*-Sinapic	BQL	2.043	3.070	5.867	7.414
RSD%	—	2.34	4.12	1.21	2.31

RSD% = relative standard deviation (*n *=* *3); BQL = peak detected, concentration lower than the LOQ but higher than the LOD.

## References

[1] Ohama H., Ikeda H., Moriyama H. (2006). Health foods and foods with health claims in Japan. *Toxicology*.

[2] Roberfroid M. B. (2007). Inulin-type fructans: functional food ingredients. *Journal of Nutrition*.

[3] Kazeem M. I., Davies T. C. (2016). Anti-diabetic functional foods as sources of insulin secreting, insulin sensitizing and insulin mimetic agents. *Journal of Functional Foods*.

[4] Jackson C. J. C., Paliyath G., Paliyath G., Bakovic M., Shetty K. (2011). Functional foods and nutraceuticals. *Functional Foods, Nutraceuticals, and Degenerative Disease Prevention*.

[5] Shin G. H., Kim J. T., Park H. J. (2015). Recent developments in nanoformulations of lipophilic functional foods. *Trends in Food Science and Technology*.

[6] Oniszczuk T., Oniszczuk A., Gondek E. (2016). Active polyphenolic compounds, nutrients contents and antioxidant capacity of extruded fish feed containing purple coneflower (*Echinacea purpurea*). *Saudi Journal of Biological Sciences*.

[7] Sowa I., Wójciak-Kosior M., Strzemski M., Rokicka K., Blicharski T., Kocjan R. (2014). Analysis of compounds with phytoestrogenic activity in dietary supplements with use of HPTLC-densitometry method. *Acta Poloniae Pharmaceutica*.

[8] Wojciak-Kosior M., Sowa I., Szymczak G., Zapała K., Kocjan R., Blicharski T. (2016). Effect of sample pre-treatment on isoflavones quantification in soybean. *Chemical Papers*.

[9] Mink P. J., Scrafford C. G., Barraj L. M. (2007). Flavonoid intake and cardiovascular disease mortality: a prospective study in postmenopausal women. *American Journal of Clinical Nutrition*.

[10] Del Rio D., Stalmach A., Calani L., Crozier A. (2010). Bioavailability of coffee chlorogenic acids and green tea flavan-3-ols. *Nutrients*.

[11] Peterson J. J., Dwyer T. J., Jacques P. F., McCullough M. L. (2012). Associations between flavonoids and cardiovascular disease incidence or mortality in European and US populations. *Nutrition Reviews*.

[12] Hooper L., Kay C., Abdelhamid A. (2012). Effects of chocolate, cocoa, and flavan-3-ols on cardiovascular health: a systematic review and meta-analysis of randomized trials. *American Journal of Clinical Nutrition*.

[13] Habauzit V., Morand C. (2012). Evidence for the cardioprotective benefits of polyphenols-containing foods: an update for clinicians. *Therapeutic Advances in Chronic Disease*.

[14] Onakpoya I. J., Spencer E. A., Thompson M. J., Heneghan C. J. (2015). The effect of chlorogenic acid on blood pressure: a systematic review and meta-analysis of randomized clinical trials. *Journal of Human Hypertension*.

[15] Gonzalez R., Ballester I., Lopez-Posadas R. (2011). Effects of flavonoids and other polyphenols on inflammation. *Critical Reviews in Food Science and Nutrition*.

[16] Karcz D., Boroń B., Matwijczuk A. (2014). Lessons from chlorophylls: modifications of porphyrinoids towards optimized solar energy conversion. *Molecules*.

[17] Kocira A., Kocira S., Złotek U., Kornas R., Świeca M. (2015). Effects of Nano-Gro preparation applications on yield components and antioxidant properties of common bean (*Phaseolus vulgaris* L.). *Fresenius Environmental. Bulletin*.

[18] Kocira A., Świeca M., Kocira S., Złotek U., Jakubczyk A. (2016). Enhancement of yield, nutritional and nutraceutical properties of two common bean cultivars following the application of seaweed extract (*Ecklonia maxima*). *Saudi Journal of Biological Sciences*.

[19] Oniszczuk A., Olech M., Oniszczuk T., Wojtunik K., Wójtowicz A. (2016). Extraction methods, LC-ESI-MS/MS analysis of phenolic compounds and antiradical properties of functional food enriched with elderberry flowers or fruits. *Arabian Journal of Chemistry*.

[20] Wójciak-Kosior M., Sowa I., Blicharski T. (2016). The stimulatory effect of strontium ions on phytoestrogens content in *Glycine max* (L.) Merr.. *Molecules*.

[21] Hykkerud Steindal A. L., Rodven R., Hansen E., Molmann J. (2015). Effects of photoperiod, growth temperature and cold acclimatisation on glucosinolates, sugars and fatty acids in kale. *Food Chemistry*.

[22] Neugart S., Klaring H. P., Zietz M. (2012). The effect of temperature and radiation on flavonol aglycones and flavonol, glycosides of kale (*Brassica oleracea* var. *sabellica*). *Food Chemistry*.

[23] Olsen H., Aaby K., Borge G. I. A. (2009). Characterization and quantification of flavonoids and hydroxycinnamic acids in curly kale (*Brassica oleracea* L. convar. *acephale* var. *sabellica*) by HPLC-DAD-ESI-MSn. *Journal of Agricultural and Food Chemistry*.

[24] Bjorkman M., Klingen I., Birch A. N. E. (2011). Phytochemicals of Brassicaceae in plant protection and human health–Influences of climate, environment and agronomic practice. *Phytochemistry*.

[25] Nyman M., Åkesson B., Nilsson J. (2006). Variation in the content of glucosinolates, hydroxycinnamic acids, carotenoids, total antioxidant capacity and low-molecular-weight carbohydrates in Brassica vegetables. *Journal of the Science of Food and Agriculture*.

[26] Brennan C., Brennan M., Derbyshire E., Tiwari B. K. (2011). Effects of extrusion on the polyphenols, vitamins and antioxidant activity of foods. *Trends in Food Science and Technology*.

[27] Oniszczuk A., Wójtowicz A., Oniszczuk T. (2015). Extruded corn gruels containing linden flowers: quantitation of phenolic compounds and selected quality characteristics. *Open Chemistry*.

[28] Wójtowicz A., Kolasa A., Mościcki L. (2013). The influence of buckwheat addition on physical properties, texture and sensory characteristic of extruded corn snacks. *Polish Journal of Food and Nutrition Science*.

[29] Oniszczuk A., Olech M. (2016). Optimization of ultrasound-assisted extraction and LC-ESI-MS/MS analysis of phenolic acids from *Brassica oleracea* L. var. *sabellica*. *Industrial Crops and Products*.

[30] Marteau C., Nardello-Rataj V., Favier D., Aubry J. M. (2013). Dual role of phenols as fragrances and antioxidants: mechanism, kinetics and drastic solvent effect. *Flavour and Fragrance Journal*.

[31] Oniszczuk A., Oniszczuk T., Wójtowicz A., Wojtunik K., Kwaśniewska A., Waksmundzka-Hajnos M. (2015). Radical scavenging activity of extruded corn gruels with addition of linden inflorescence. *Open Chemistry*.

[32] Bouasla A., Wójtowicz A., Zidoune M. N. (2016). Gluten-free precooked rice-yellow pea pasta: effect of extrusion-cooking conditions on phenolic acids composition, selected properties and microstructure. *Journal of Food Science*.

[33] Schmidt S., Zietz M., Schreiner M., Rohn S., Kroh L. W., Krumbein A. (2010). Genotypic and climatic influences on the concentration and composition of flavonoids in kale (*Brassica oleracea* var. *sabellica*). *Food Chemistry*.

[34] Grace M. H., Yousef G. G., Esposito D., Raskin I., Lila M. A. (2014). Bioactive capacity, sensory properties, and nutritional analysis of a shelf stable protein-rich functional ingredientwith concentrated fruit and vegetable phytoactives. *Plant Foods for Human Nutrition*.

[35] Esposito D., Chen A., Grace M., Komarnytsky S., Lila M. (2014). Inhibitory effects of wild blueberry anthocyanins and other flavonoids on biomarkers of acute and chronic inflammation in vitro. *Journal of Agricultural and Food Chemistry*.

[36] Anton A. A., Fulcher R. G., Arntfield S. D. (2009). Physical and nutritional impact of fortification of corn starch-based extruded snacks with common bean (*Phaseolus vulgaris* L.) flour: effects of bean addition and extrusion cooking. *Food Chemistry*.

[37] Korus J., Gumul D., Czechowska K. (2007). Effect of extrusion on the phenolic composition and antioxidant activity of dry beans of *Phaseolus vulgaris* L.. *Food Technology and Biotechnology*.

[38] Dlamini N. R., Taylor J. R. N., Rooney L. W. (2007). The effect of sorghum type and processing on the antioxidant properties of African sorghum-based foods. *Food Chemistry*.

[39] Zielinski H., Michalska A., Piskula M. K., Kozlowska H. (2006). Antioxidants in thermally treated buckwheat groats. *Molecular Nutrition & Food Research*.

